# *Ginkgobiloba* leaf extract mitigates cisplatin-induced chronic renal interstitial fibrosis by inhibiting the epithelial-mesenchymal transition of renal tubular epithelial cells mediated by the Smad3/TGF-β1 and Smad3/p38 MAPK pathways

**DOI:** 10.1186/s13020-022-00574-y

**Published:** 2022-02-21

**Authors:** Congying Wei, Yansong Zhang, Xiaobin Zhong, Sisi Lu, Xiaoqin Zou, Yufang Yang, Songqing Huang, Zhenguang Huang

**Affiliations:** 1 Department of Pharmacy, The First Affiliated Hospital of Guangxi Medical University, 6 Shuangyong Road, Nanning, 530021 Guangxi Zhuang Autonomous Region People’s Republic of China; 2 Regenerative Medicine Research Center of Guangxi Medical University, Shuangyong Road, Nanning, 530022 Guangxi Zhuang Autonomous Region People’s Republic of China

**Keywords:** *Ginkgo biloba* leaf extract, Cisplatin, Renal interstitial fibrosis, Epithelial-mesenchymal transition, Smad3/TGF- β1, Smad3/p38 MAPK pathway

## Abstract

**Background:**

Our previous study indicated that *Ginkgo biloba* leaf extract (EGb) could protect against cisplatin-induced acute kidney injury in rabbits. The present study aimed to determine the effects and potential molecular mechanisms of EGb on chronic renal interstitial fibrosis induced by cisplatin using in vivo and in vitro models.

**Methods:**

Rats received a single dose of cisplatin on Day 1, and a subset of rats was intraperitoneally injected with EGb daily between Days 22–40. In vitro, HK-2 cells were treated with cisplatin, and a subset of cells was cultivated with EGb or SIS3 (Smad3 inhibitor) for 48 h. Renal function of rats was assessed by detecting the levels of serum creatinine (Scr), blood urea nitrogen (BUN) and urinary N-acetyl-β-D-glucosaminidase (NAG). Hematoxylin and eosin staining and Masson’s trichrome staining were used to evaluate the damage and fibrosis of renal tissue. Western blotting, immunohistochemistry and immunofluorescence were used to detect the protein levels of fibrosis-associated proteins and signaling pathway-related proteins. RT–qPCR analysis was used to examine the mRNA levels of related indicators.

**Results:**

EGb significantly decreased the increased levels of Scr, BUN and urinary NAG and attenuated renal damage and the relative area of renal interstitial fibrosis induced by cisplatin. Additionally, EGb decreased the protein levels of α-SMA, Col I, TGF-β1, smad2/3, phosphorylated (p)-smad2/3, p38 MAPK, and p-p38 MAPK; the ratio of p-p38 MAPK/p38 MAPK; and the mRNA level of *p38 MAPK* in renal tissues induced by cisplatin. In agreement with in vivo studies, EGb significantly reduced the increased protein levels of these indicators. Additionally, EGb significantly reduced the increased protein levels of vimentin, TIMP-1, and CTGF, as well as the mRNA levels of α-SMA, vimentin, and *TGF-β1*, while it significantly increased the reduced E-cadherin protein level and the MMP-1/TIMP-1 ratio in HK-2 cells induced by cisplatin. It’s worth noting that the effects of SIS3 in changing the above indicators were similar to those of EGb.

**Conclusion:**

Our study demonstrated that EGb improved cisplatin-induced chronic renal interstitial fibrosis, and its mechanisms were associated with inhibiting the epithelial-mesenchymal transition of renal tubular epithelial cells via the Smad3/TGF-β1 and Smad3/p38 MAPK pathways.

**Supplementary Information:**

The online version contains supplementary material available at 10.1186/s13020-022-00574-y.

## Introduction

Cisplatin is a major anticancer agent used widely for the treatment of various types of tumors [1]. However, acute kidney injury is a severe side effect that limits the clinical use of cisplatin [2]. Cisplatin-induced acute kidney injury mainly manifests as damage to the renal tubulointerstitium and eventually develops into fibrosis [3]. Regardless of the primary disease process, interstitial fibrosis is considered to be the key determinant of irreversible renal failure [4]. Therefore, it is important to identify agents that can limit the process of cisplatin-induced renal interstitial fibrosis (CRIF).

Our previous study demonstrated that cisplatin-induced acute kidney injury may develop into chronic renal interstitial fibrosis [5]. However, the mechanisms of the occurrence and development of chronic renal interstitial fibrosis induced by cisplatin have not been clarified. According to reports, epithelial-mesenchymal transition (EMT) of renal tubular epithelial cells is one main mechanism of CRIF [6]. Transforming growth factor β1 (TGF-β1), which is a potent fibrogenic factor and plays a crucial role in the development of renal fibrosis [7, 8], could promote the cisplatin-induced EMT of NRK-52E [9] and exert its biological functions via the p38 mitogen-activated protein kinase (MAPK) pathway in hepatitis B virus-induced tubular EMT [10]. Additionally, TGF-β1/Smad induced EMT of renal tubular epithelial cells and promoted renal fibrosis in rats after subtotal nephrectomy [11]. However, the role and the relationship of TGF-β1, p38 MAPK and Smads in the cisplatin-induced EMT of renal tubular epithelial cells and CRIF are still unclear.

*Ginkgo biloba* leaf extract (EGb) is produced from *G. biloba* leaves, which is rich in compounds used as dietary supplements and in beverages [12, 13] and has been used in traditional Chinese medicine for the prevention and treatment of Alzheimer’s disease and cognitive deficits [14, 15]. Previous studies have reported that EGb suppresses hepatic fibrosis through inhibition of the TGF-β1 or p38 MAPK pathways [16, 17]. EGb treatment has also been reported to reduce the level of phosphorylated (p)-p38 MAPK and to attenuate brain death-induced kidney injury in a rat model [18]. A previous report noted that EGb has a beneficial effect on cisplatin-induced renal failure in rats [5]. Our previous studies demonstrated that EGb exerts a protective effect against cisplatin-induced acute kidney injury in rabbits [19] and alleviates CRIF in rats through inhibition of apoptosis via regulation of the p38 MAPK/TGF-β1 and p38 MAPK/HIF-1α pathways [5]. However, the effects and mechanisms of EGb on the cisplatin-induced EMT of renal tubular epithelial cells, as well as the role and relationship of p38 MAPK, TGF-β1 and Smads in this process, remain to be elucidated. Therefore, the present study aimed to explore these issues using rat models with CRIF and HK-2 EMT induced by cisplatin.

## Materials and methods

### Materials

EGb (Batch No. IB122) was provided by Dr. Willmar at Schwabe Pharmaceuticals. Each 5-ml ampule contained 17.5 mg of EGb, which contained 24% ginkgo flavonol glycosides and 6% terpene lactones in ethanol. Powder injection of cisplatin (Batch No. 5050272DB), dissolved in normal saline before use, was purchased from Qilu Pharmaceutical Co., Ltd. SIS3 (Smad3 inhibitor) was purchased from MCE (HY-13013, Shanghai, China). Minimum essential medium (MEM) (batch no. WH01122101XP01) was purchased from Procell (PM150410, Wuhan, China). Antibodies against α-smooth muscle actin (α-SMA; cat. no. BM0002), type I collagen (Col I; cat. no. BA0325), TIMP-1 (cat. no. D10E6), MMP-1 (cat. no. E9S9N), CTGF (cat. no. D8Z8U), TGF-β1 (cat. no. ab179695), p38 MAPK (cat. no. 9215S), p-p38 MAPK (cat. no. 8690S) and p-smad2/3 (cat. no. 8828S) were obtained from Cell Signaling Technology, Inc.; antibodies against vimentin (cat. no. 10366-1-AP), E-cadherin (cat. no. 20874-1-AP) and beta-actin (β-actin; cat. no. 20536-1-AP) were obtained from Proteintech; and an antibody against GAPDH (cat. no. ab181602) was purchased from Abcam.

### Animals

Male Sprague–Dawley rats (weight, 200 ± 20 g; age, 7 weeks) were obtained from the Experimental Animal Center of Guangxi Medical University (Guangxi, China). The research was conducted according to protocols approved by the Institutional Ethics Committee of Guangxi Medical University (Approval No. 201310009). The rats were housed in an air-conditioned room at 25 ± 2 °C with an estimated 60 ± 10% relative humidity. The animals were maintained on a 12-h light–dark cycle and had ad libitum access to water and food.

### Experimental design

Following 1 week of acclimatization, the rats were randomly divided into five groups (n  = 9 rats/group) as presented in Table 1. All drugs were administered via intraperitoneal injection. The dose of EGb in the cisplatin  +  M-EGb group was converted from the usual clinical dose of adult [20]. The rats in the cisplatin-treated groups received a single dose of cisplatin on Day 1 to induce renal interstitial fibrosis [21, 22].

**Table 1:** Experimental design and drug treatment

Group	Drug (dose)	
	Day 1	Day 22–40
Control	Equal volume of saline	Saline equal volume to that of EGb (3.17 mg/kg/day)
Cisplatin	Cisplatin (5 mg/kg)	Saline equal volume to that of EGb (3.17 mg/kg/day)
Cisplatin + L-EGb	Cisplatin (5 mg/kg)	EGb (1.58 mg/kg/day)
Cisplatin + M-EGb	Cisplatin (5 mg/kg)	EGb (3.17 mg/kg/day)
Cisplatin + H-EGb	Cisplatin (5 mg/kg)	EGb (6.34 mg/kg/day)

*L* low dose; *M* medium dose; *H* high dose; *EGb* *Ginkgo* *biloba* leaf extract

### Cell culture

Human renal tubular epithelial (HK-2) cells were purchased from Procell (Wuhan, China) and cultivated in MEM supplemented with 10% fetal bovine serum (FBS) at 37 ℃ in a 5% CO_2_ incubator. HK-2 cells were divided into five groups as follows: a Control cell group (HK-2 cells were cultivated in MEM with 10% FBS), a Model cell group (cells were cultivated in MEM with 10% FBS, 2 µg/ml cisplatin), an EGb group (cells were cultivated in MEM with 10% FBS, 2 µg/ml cisplatin and 350 µg/ml EGb), an SIS3 group (cells were cultivated in MEM with 10% FBS, 2 µg/ml cisplatin and 5.0 μM E-SIS3), and an EGb + EIS3 group (cells were cultivated in MEM with 10% FBS, 2 µg/ml cisplatin, 5.0 μM E-SIS3 and 350 µg/ml EGb).

### Specimen collection of blood, urine and renal tissues

At the end of each treatment (12 h following the last treatment on Day 40), blood, urine and renal tissue samples were collected and stored at − 80 °C for future analysis. Briefly, after urine samples were obtained, rats were anesthetized intraperitoneally with sodium pentobarbital (30 mg/kg), and blood samples (5 ml/per rat) were collected from the abdominal aorta and centrifuged at 1409×*g* for 15 min at 4 °C. The rats were euthanized by exsanguination under deep anesthesia. When the rats exhibited no breathing or reflexes, the renal tissues were collected and washed with ice-cold saline, and some of the renal tissues (corticomedullary junction) were fixed in 10% buffered formalin for hematoxylin and eosin (H&E), Masson’s trichrome and immunohistochemical staining. The other tissues were stored immediately at − 80 °C for western blot and reverse transcription-quantitative (RT-q) PCR analyses.

### Determination of blood urea nitrogen (BUN), serum creatinine (Scr) and urinary N-acetyl-β-D-glucosaminidase (NAG) levels

BUN (cat. no. C013-2), Scr (cat. no. C011-1) and urinary NAG (cat. no. A031) levels were determined using the corresponding assay kits obtained from Nanjing Jiancheng Bioengineering Institute according to the manufacturer’s instructions. The levels of BUN and Scr were measured with a 7100 automatic biochemical analyzer (Hitachi, Ltd.). The level of urinary NAG was detected using the nitrophenol colorimetric method with a continuous spectrum scanning microplate reader (Spectra Maxplus 384; Molecular Devices, LLC) at a wavelength of 400 nm.

### H&E staining

Renal samples were fixed with 10% buffered formalin and embedded in paraffin. Subsequently, the renal tissues were sectioned into 3–4-mm slices and stained with H&E. The sections were examined under an Olympus IX51 light microscope (Olympus Corporation). The tubule interstitial injury scores were determined according to a previous study [23] as follows: 0 points, no damage; 1 point,  < 25% damage; 2 points, 25–50% damage; and 3 points,  > 50% damage. A total of five randomly selected fields (× 400 magnification) were evaluated per specimen, and the mean score was calculated. Histopathological injury was scored by an experienced pathologist blinded to the experimental conditions.

### Masson’s trichrome staining

The aforementioned formalin-fixed sections of renal tissues were stained with Masson’s trichrome to evaluate the fibrotic areas. Five randomly selected fields (× 400) were evaluated for each specimen using a light microscope, and the average of the relative area of interstitial fibrosis was assessed using a Color Image Analyzer (Image-Pro Plus 6.0; Media Cybernetics, Inc.) [24]. The areas overlaying the tubular basement membrane and interstitial space were evaluated, whereas the glomeruli and large vessels were excluded from the analysis. The histological examinations were performed by an experienced pathologist blinded to the experimental conditions.

### Immunohistochemical detection of α-SMA, Col I and TGF-β1 protein expression in renal tissue

Briefly, renal samples were embedded in paraffin, sectioned (3 µm), dewaxed, dehydrated (sections were incubated sequentially for 5 min in anhydrous ethanol, 5 min in 95% ethanol and 5 min in 75% ethanol) and immersed in 0.3% H_2_O_2_ for 10 min at room temperature to block endogenous peroxidase activity. The samples were incubated with 10% goat serum (Beijing Zhongshan Jinqiao Biotechnology Co., Ltd.) at room temperature for 15 min to block nonspecific binding. Subsequently, the sections were incubated with rabbit anti-rat primary antibodies against α-SMA (1:6000), Col I (1:1400) and TGF-β1 (1:6000) overnight at 4 °C. The samples were subsequently incubated with a biotinylated secondary antibody (biotin-labeled goat anti-rabbit IgG; Beijing Zhongshan Jinqiao Biotechnology Co., Ltd.; cat. no. WP151228; 1:100) for 1 h at 37 °C, and color development was induced using a 3′3′-diaminobenzidine (DAB) kit (Wuhan Boster Biological Technology, Ltd.). Finally, the renal samples were visualized using a light microscope (Olympus Soft Imaging Solutions GmbH). The positively stained areas were quantified by integrated optical density (IOD) analysis using Image-Pro Plus 6.0 software. A total of 10 randomly selected fields (× 400 magnification) were assessed for each specimen, and the mean value was calculated. The protein expression levels of α-SMA, Col I and TGF-β1 are presented as the ratio of IOD/positively stained areas to unstained tissues.

### Immunofluorescence analysis

HK-2 cells were collected and fixed with 4% formaldehyde for 30 min. The cells were penetrated with 0.5% Triton X-100 for 20 min at room temperature. Cells were blocked with goat serum for 30 min at room temperature and then incubated with primary antibodies overnight at 4 °C at the indicated dilutions (1:100 for E-cadherin, α-SMA, TGF-β1 and vimentin; 1:50 for p-smad2/3). The coverslips were then washed and incubated with fluorescein-labeled secondary antibody for 1 h at room temperature in the dark. Finally, the cells were counterstained with DAPI and examined under a fluorescence microscope (OLYMPUS BX53, Japan). The image J software was used to measure the fluorescence intensity of the positive area. A total of five randomly selected fields (× 400 magnification) were evaluated for each sample, and the average fluorescence intensity under each field was calculated.

### Western blotting detection of α-SMA, E-cadherin, vimentin, COL-I, TIMP-1, MMP-1, CTGF, p38 MAPK, p-p38 MAPK, smad2/3, p-smad2/3 and TGF-β1

Kidney tissues from rats in different groups were homogenized with RIPA lysis buffer (Beyotime Institute of Biotechnology). The protein lysates were quantified using a BCA protein assay kit (Beyotime Institute of Biotechnology) according to the manufacturer’s instructions. Equal amounts (500 μg per lane) of protein lysates were separated by 12% SDS–PAGE (100 V, 2 h), and the proteins were then transferred to PVDF membranes (EMD Millipore) at 100 V for 1.5 h at 4 °C. The membranes were blocked with 5% QuickBlock™ Western (cat. no. P0252; Beyotime Institute of Biotechnology) at room temperature for 1 h, followed by incubation with rabbit anti-rat primary antibodies against α-SMA (1:1000), E-cadherin (1:3000), vimentin (1:4000), COL-I (1:500), TIMP-1 (1:1000), MMP-1 (1:1000), CTGF (1:1000), p38 MAPK (1:500), p-p38 MAPK (1:1,000), p-smad2/3 (1:1000), smad2/3 (1:1000) and anti-TGF-β1 (1:500) overnight at 4 °C. Following washing, the membranes were incubated using a secondary fluorescent goat anti-rabbit antibody (1:10,000; cat. no. 5151S; Cell Signaling Technology, Inc.) for 1 h and were then detected using the Odyssey infrared fluorescence scanning imaging system 3.0 (LI-COR Biosciences, USA). GAPDH (1:1000) and β-actin (1:2000) were used as internal controls.

### RT–qPCR analysis for α-SMA, vimentin, TGF-β1, and p38 MAPK mRNA detection

Total RNA was extracted from renal tissues and HK-2 cells with TRIzol^®^ reagent (Invitrogen; Thermo Fisher Scientific, Inc.) according to the manufacturer’s instructions. Total RNA (1 µg) was reverse-transcribed using the Prime Script™ RT reagent kit with gDNA Eraser (Takara Bio, Inc.). For renal samples, the program included denaturation at 95 °C for 30 s, 40 cycles of 95 °C for 5 s and 60 °C for 31 s; for cell samples, the program included denaturation at 37 ℃ for 15 min, 85 ℃ for 5 s and end at 4 ℃. Following extraction, qPCR was performed using a SYBR^®^ Premix Ex Taq™ II kit (Takara Bio, Inc.) according to the manufacturer’s instructions and on an Applied Biosystems 7500 Real-Time PCR System (Applied Biosystems; Thermo Fisher Scientific, Inc.). The PCR primers used are shown in Table 2. The thermocycling conditions for renal samples were as follows: 95 °C for 30 s, followed by 40 cycles of 95 °C for 5 s and 60 °C for 34 s; the expression level of *p38 MAPK* was normalized to that of GAPDH using the 2^−ΔΔCq^ method, in which ΔΔCq = (Cq_target_ − Cq_GAPDH_) sample − (Cq_target_ − Cq_GAPDH_) control [25]. The thermocycling conditions for cell samples were 95 °C for 5 min, 40 cycles of 95 °C for 15 s and 60 °C for 1 min, and the expression was determined by the 2^−ΔΔCq^ method, wherein ΔΔCq  =  (Cq_target_ − Cq_β-actin_) sample − (Cq_target_ − Ct_β-actin_) control.

**Table 2:** The list of prisms used in RT-PCR for mRNA expression

Gene	Forward primer (5′–3′)	Reverse primer (5′–3′)
α-SMA	AGGAGCAAAATCTGTCCGATCT	GTGGGGGAATTATCTTTCCTGG
Vimentin	TGAATGACCGCTTCGCCAACTAC	CTCCCGCATCTCCTCCTCGTAG
TGF-β1	ACCTCGGCTGGAAGTGG	CCGGGTTATGCTGGTTGT
p38MAPK	TTACCGATGACCACGTTCAGTTTC	AGCGAGGTTGCTGGGCTTTA
GAPDH	GGCACAGTCAAGGCTGAGAATG	ATGGTGGTGAAGACGCCAGTA
β-actin	CTACCTCATGAAGATCCTCACCGA	TTCTCCTTAATGTCACGCACGATT

### Statistical analysis

Data are presented as the mean  ±  SD. Statistical analysis was performed using SPSS 20.0 software for Windows (IBM Corp.). One-way analysis of variance (One-way ANOVA) was used for comparison between multiple groups. LSD was used for pairwise comparison of variance, and Dunnett’s T3 method was used for pairwise comparison of uneven variance. P  < 0.05 was considered to indicate a statistically significant difference.

## Results

### EGb reduces the levels of BUN, Scr and urinary NAG in rats treated with cisplatin

**Fig. 1:**
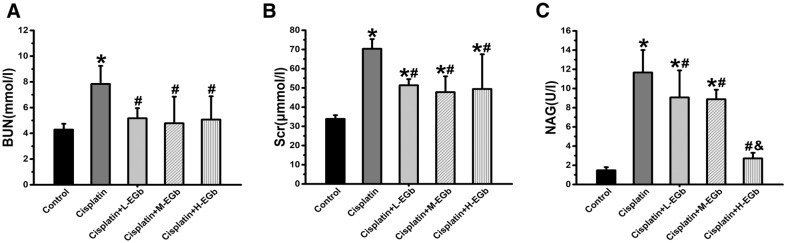
The levels of **A** BUN, **B** Scr and **C** urinary NAG levels in rats. n  = 9. *P  < 0.05 vs. control; ^#^P  < 0.05 vs. cisplatin; ^&^P  < 0.05 vs. cisplatin  +  M-EGb. *BUN* blood urea nitrogen; *Scr* serum creatinine; *NAD* N-acetyl-β-D-glucosaminidase; *L* low-dose; *M* medium-dose; *H* high-dose; *EGb* *Ginkgo biloba* leaf extract

As demonstrated in Fig. 1, the levels of BUN, Scr and urinary NAG were significantly increased in the cisplatin-only group compared with the control group. The levels of BUN were significantly decreased in these EGb-treated groups compared with the cisplatin-only group (Fig. 1A). The levels of Scr were significantly decreased in these EGb-treat groups compared with the cisplatin-only group (Fig. 1B), and the levels of urinary NAG were significantly decreased in the cisplatin  +  H-EGb group compared with the cisplatin-only group and the cisplatin  +  M-EGb group (Fig. 1C).

### EGb reduces cisplatin-induced renal tissue damage and renal interstitial fibrosis

**Fig. 2:**
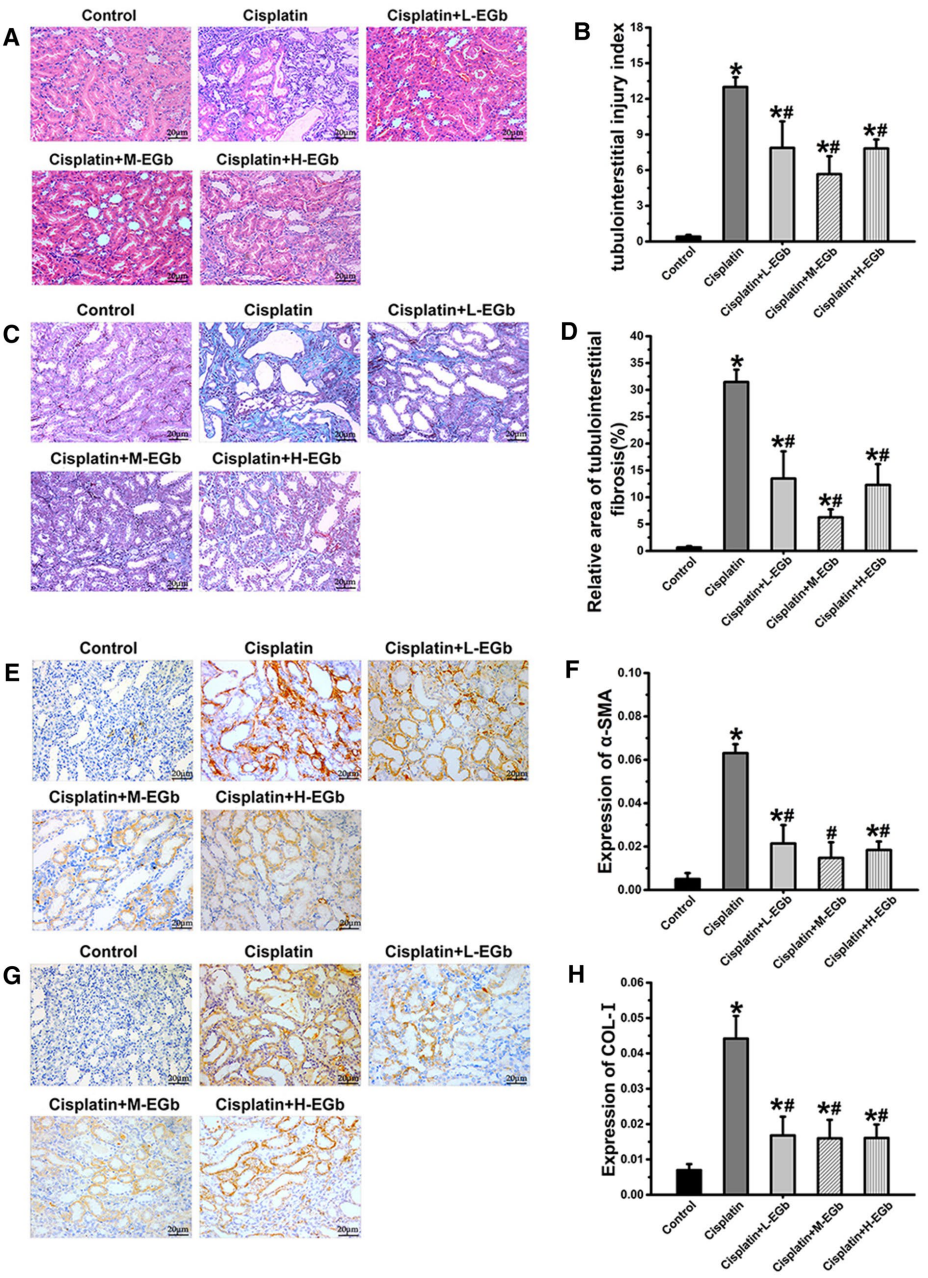
The effect of EGb on cisplatin-induced renal fibrosis in rats. **A** Representative images (× 400 magnification) of H&E-stained renal tissue samples. **B** Tubulointerstitial injury indices of rats in the indicated groups. **C** Representative images (× 400 magnification) of Masson’s trichrome-stained renal tissue samples. **D** Relative area of tubulointerstitial fibrosis (%) in rats in the indicated groups. **E**, **G** Representative images (× 400 magnification) of α-SMA and Col I immunohistochemically stained renal tissue sections. Quantification the protein levels of **F** α-SMA and **H** Col I in rat renal tissues. n  = 9. *P  < 0.05 vs. control; ^**#**^P  < 0.05 vs. cisplatin; ^&^P  < 0.05 vs. cisplatin  +  M-EGb. *α-SMA* α-smooth muscle actin; *Col I* type I collagen; *L* low-dose; *M* medium-dose; *H* high-dose; *EGb* *Ginkgo* *biloba* leaf extract

H&E analysis revealed no histopathological damage to the renal tissues obtained from the control group. Cisplatin treatment resulted in renal tubular lumen stenosis, and a number of renal tubular epithelial cells presented signs of edema, degeneration or necrosis compared with the control group (Fig. 2A). In contrast, EGb treatment alleviated the damage to renal tissues induced by cisplatin (Fig. 2A). A higher tubular injury score was observed in the cisplatin-only group than in the control group. In contrast, the tubular injury score was significantly reduced in these EGb treatment groups compared with the cisplatin-only group (Fig. 2B).

MASSON analysis revealed that the renal tissues in the control group exhibited no renal fibrogenesis, whereas cisplatin treatment resulted in notable renal interstitial fibrogenesis (Fig. 2C). The relative area of renal interstitial fibrosis was significantly higher in the cisplatin-only group than in the control group. However, the relative area of renal interstitial fibrosis was significantly reduced in these EGb-treated groups compared with the cisplatin-only group (Fig. 2D).

Additionally, immunohistochemical analysis showed that the α-SMA and Col I proteins were mainly expressed in renal tubular epithelial cells and interstitial cells (Fig. 2E, G). The protein levels of α-SMA and Col I were significantly increased in the cisplatin-only group compared with the control group, while the protein levels of α-SMA and Col I were significantly reduced in these EGb-treated groups compared with the cisplatin-only group (Fig. 2F, H).

### EGb reduces the levels of TGF-β1 in renal tissues from rats treated with cisplatin

**Fig. 3:**
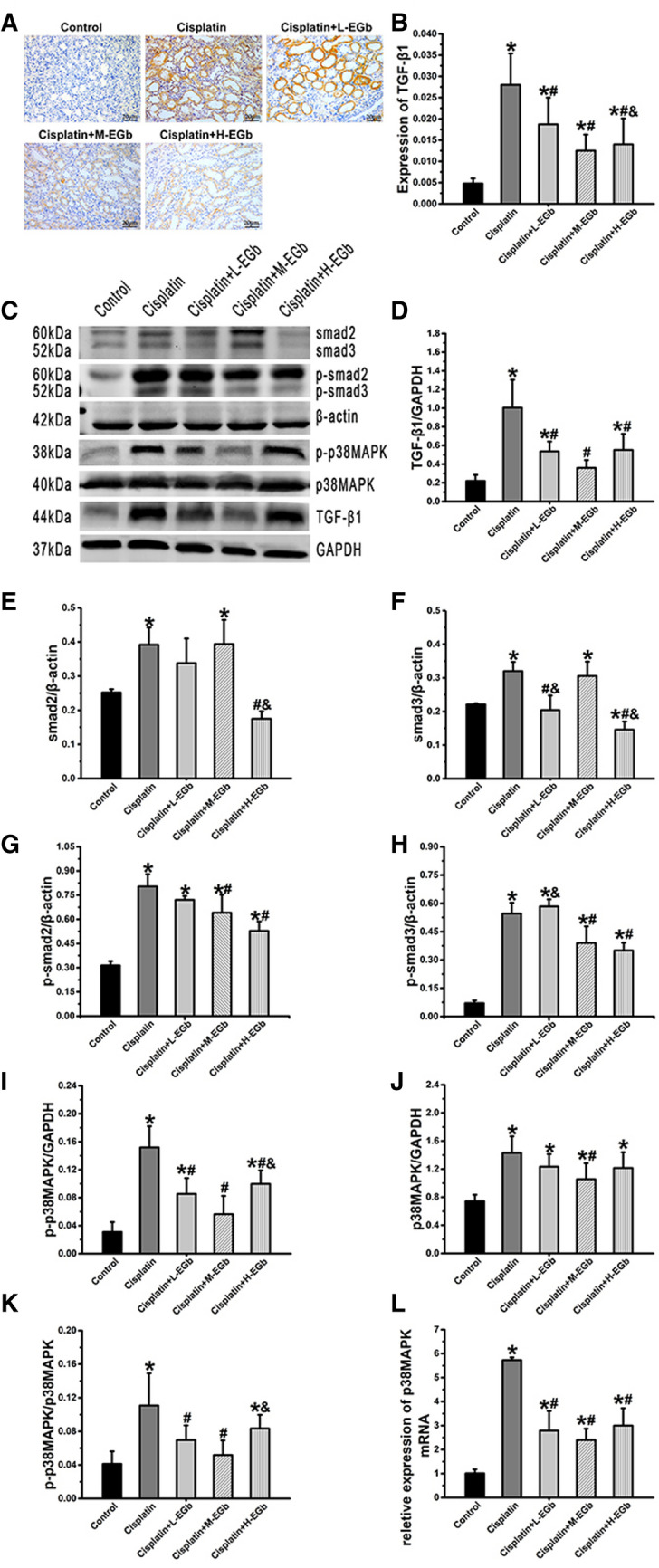
The effect of EGb on the protein expression levels of TGF-β1, smad2/3, p-smad2/3, p-p38MAPK, p38MAPK and the effect of p38 MAPK gene expression in rat kidney tissue. **A** Representative images (× 400 magnification) of TGF-β1 immunohistochemically stained renal tissue sections. **B** Quantification of TGF-β1 expression in renal tissues. **C** Western blot analysis of smad2/3, p-smad2/3, p-p38 MAPK, p38MAPK, TGF-β1 expression in rat kidney tissues. Quantitative analysis the protein levels of **D** TGF-β1, **E**, **F** smad2/3, **G**, **H** p-smad2/3, **I** p-p38MAPK, **J** p38MAPK, and the ratio of **K** p-p38MAPK/p38MAPK in renal tissues. **L** Reverse transcription-quantitative PCR analysis p38 MAPK mRNA expression in rat kidney tissues. n  = 9. *P  < 0.05 vs. control; ^#^P  < 0.05 vs. cisplatin; ^&^P  <  0.05 vs. cisplatin  +  M-EGb. *MAPK* mitogen-activated protein kinase; *p-* phosphorylated; *TGF-β1* transforming growth factor β1; *L* low-dose; *M* medium-dose; *H* high-dose; *EGb* *Ginkgo* *biloba* leaf extract

Immunohistochemical analysis demonstrated that the TGF-β1 protein was mainly expressed in renal tubular epithelial cells (Fig. 3A). Immunohistochemical analysis and western blot analysis revealed that the protein level of TGF-β1 was significantly increased in the cisplatin-only group compared with the control group (Fig. 3B, D). Additionally, immunohistochemical and western blot analyses indicated that the TGF-β1 level was significantly decreased in these EGb-treated groups compared with the cisplatin-only group (Fig. 3B, D).

### EGb reduces the levels of smad2/3, p-smad2/3, p38 MAPK, p-p38 MAPK and the p-p38 MAPK/p38 MAPK ratio in renal tissues from rats treated with cisplatin

Western blot analysis revealed that the protein levels of smad2/3, p-smad2/3, p38 MAPK and p-p38 MAPK, as well as the ratio of p-p38 MAPK/p38 MAPK, were significantly increased in the cisplatin-only group compared with the control group. Additionally, the protein levels of smad2/3, p-smad2/3, p-p38 MAPK, and the ratio of p-p38 MAPK/p38 MAPK were significantly decreased in these EGb-treated groups compared with the cisplatin-only group (Fig. 3). The protein levels of smad2/3 were significantly decreased in the cisplatin  +  H-EGb group compared with the cisplatin  +  M-EGb group (Fig. 3E, F). The protein level of p38 MAPK was significantly reduced in the cisplatin  +  M-EGb group compared with the cisplatin-only group (Fig. 3J).

RT–qPCR analysis demonstrated that the *p38 MAPK* mRNA level in renal tissues was significantly increased in the cisplatin-only group compared with the control group. Additionally, the *p38 MAPK* mRNA level was significantly reduced in these EGb-treated groups compared with the cisplatin-only group (Fig. 3L).

### Effect of EGb on the morphology of HK-2

**Fig. 4:**
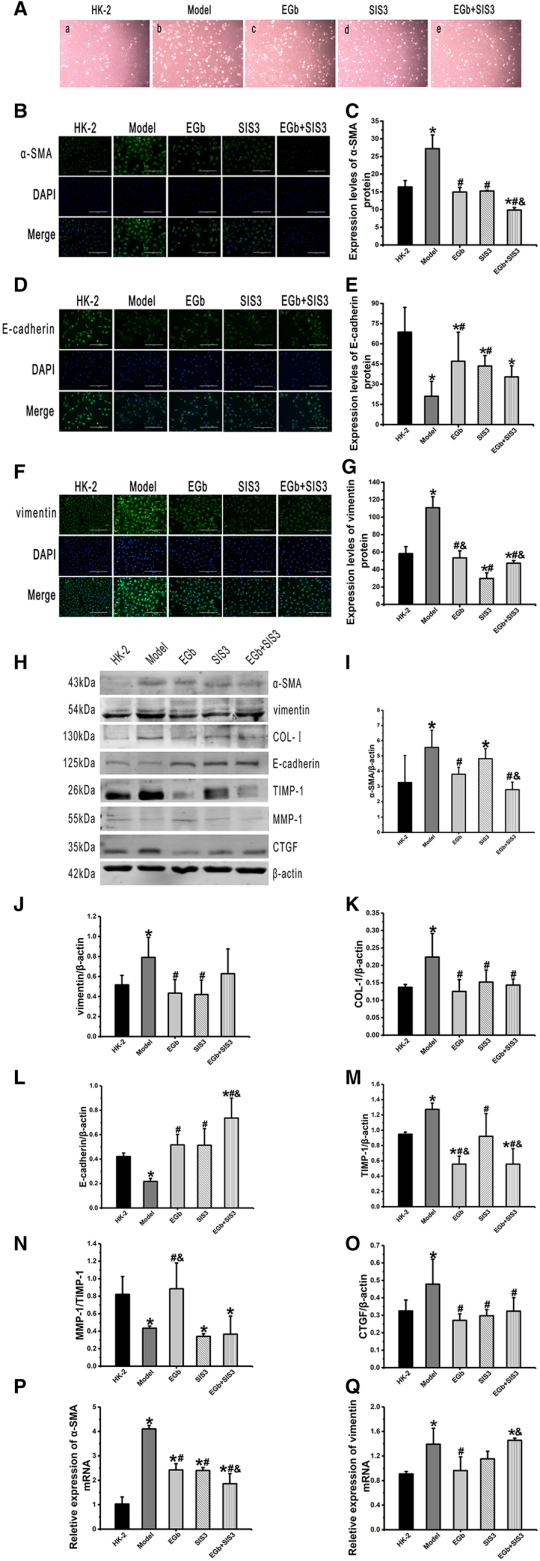
Cell morphology was observed at 400 magnification and EGb on EMT of HK-2 cells treated by cisplatin. **a** HK-2 group; **b** Model group; **c** EGb group; **d** SIS3 group; **e** EGb  +  SIS3 group. Representative immunohistochemical images of α-SMA (**B**), E-cadherin (**D**), Vimentin (**F**) in different groups of HK-2 cells. Nuclei were counterstained with DAPI (blue). Scale bar: 200 μm. Quantitative analysis of **C** α-SMA, **E** E-cadherin and **G** Vimentin expression in HK-2 cells. **H** Western blot analysis of α-SMA, vimentin, COL-I, E-cadherin, TIMP-1, MMP-1, CTGF expression in HK-2 cells. Quantitative analysis of the levels of **I** α-SMA, **J** vimentin, **K** COL-I, **L** E-cadherin, **M** TIMP-1, **N** MMP-1/TIMP-1, **O** CTGF in HK-2 cells. Reverse transcription-quantitative PCR analysis **P** α-SMA and **Q** vimentin mRNA expression in HK-2 cells. n  = 3. *P  < 0.05 vs. HK-2; ^#^P  < 0.05 vs. Model; ^&^P  < 0.05 vs. SIS3

The cells in the HK-2 group were irregular islands with cells covering the whole microscope field, precise connections, almost no gaps between cells, and a wide range of cells fused to grow into sheets or laminated growth (Fig. 4a). The cell morphology was irregular, with obvious atrophy in the field of vision, and the number of cells was significantly reduced in the model group compared with the HK-2 group (Fig. 4b). Some cells in the EGb, SIS3 and EGb  +  SIS3 groups were island-shaped with a clear outline, and the number of cells was greater than that in the model group (Fig. 4c–e).

### Effects of EGb on the levels of α-SMA, vimentin, COL-I, E-cadherin, TIMP1, MMP-1, and CTGF in HK-2 cells injured by cisplatin

As presented by immunofluorescence staining and western blot analysis, the protein levels of α-SMA and vimentin were significantly increased, while the protein level of E-cadherin was significantly decreased in the model group compared with the HK-2 group. Conversely, the protein levels of α-SMA in the EGb and EGb  +  SIS3 groups and vimentin in the EGb and SIS3 groups were decreased, while the protein levels of E-cadherin were increased in the EGb, SIS3 and EGb  +  SIS3 groups compared with the model group (Fig. 4). Additionally, western blot analysis also revealed that the protein level of α-SMA was significantly decreased, while the protein level of E-cadherin was significantly increased in the EGb  +  SIS3 group compared with the SIS3 group (Fig. 4I, L).

Western blot analysis also confirmed that the protein levels of COL-I, TIMP1, and CTGF were significantly increased, while the MMP-1/TIMP1 ratio was significantly decreased in the model group compared with the HK-2 group. Conversely, the protein levels of COL-I and CTGF in the EGb, SIS3 and EGb  +  SIS3 groups and TIMP1 in the EGb and EGb  +  SIS3 groups were significantly reduced, while the MMP-1/TIMP-1 ratio was significantly increased in the EGb group compared with the model group (Fig. 4). The protein level of TIMP-1 was significantly decreased in the EGb and EGb  +  SIS3 groups compared with the SIS3 group (Fig. 4M).

RT–qPCR analysis demonstrated that the α-SMA and vimentin mRNA expression levels were significantly increased in the model group compared with the HK-2 group (Fig. 4P, Q). Conversely, the α-SMA mRNA levels were significantly decreased in the EGb, SIS3 and EGb  +  SIS3 groups compared with the model group (Fig. 4P). Additionally, the vimentin mRNA level was significantly decreased in the EGb group compared with the model group (Fig. 4Q).

### Effects of EGb on the levels of TGF-β1, smad2/3, p-smad2/3, p38 MAPK, and p-p38 MAPK in HK-2 cells injured by cisplatin

**Fig. 5:**
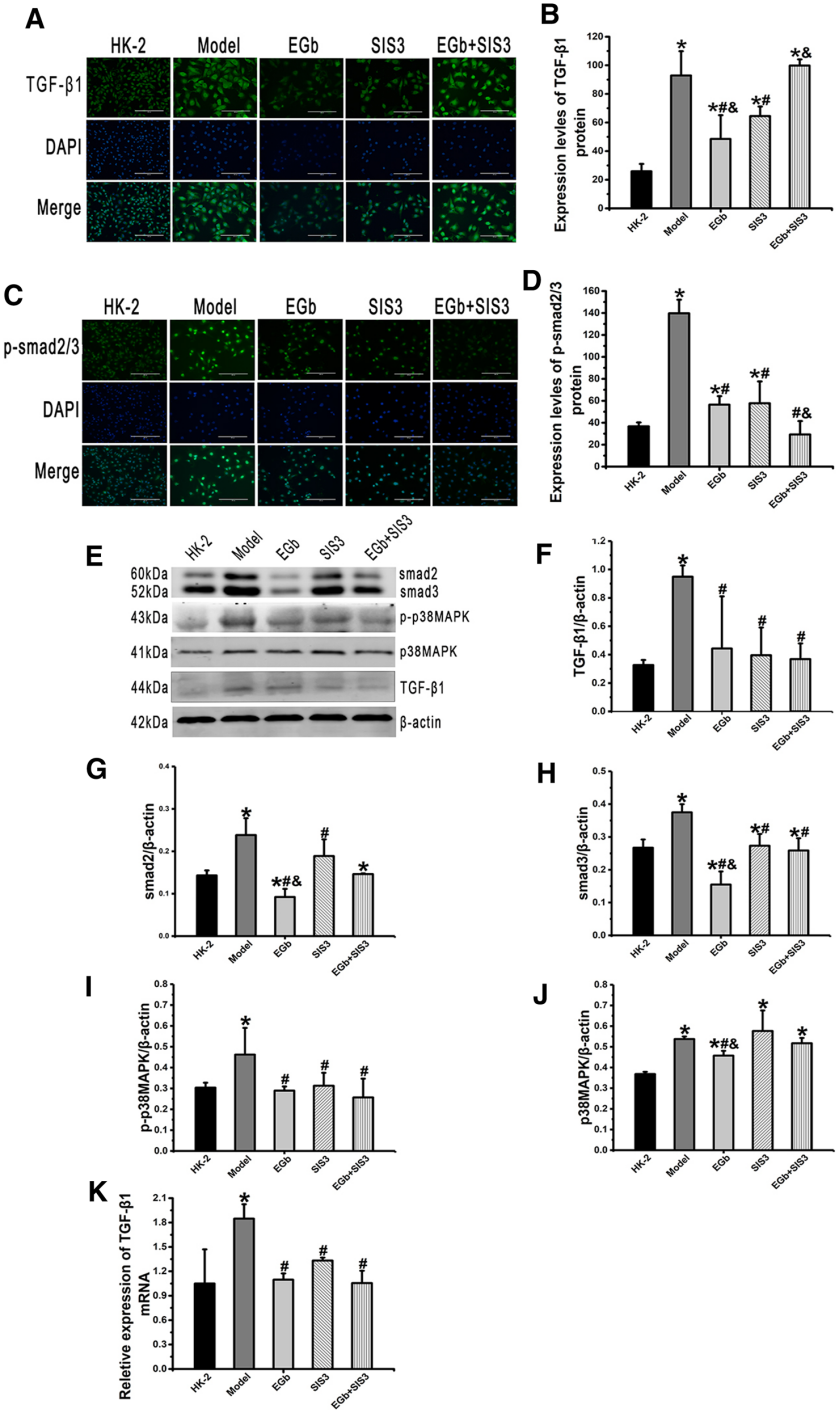
The effect of EGb on the protein levels of TGF-β1, p-smad2/3, smad2/3, p-p38MAPK and p38 MAPK, and *TGF-β1* mRNA level in HK-2 cells. Representative images of immunofluorescence staining of **A** TGF-β1, **C** p-smad2/3 in different groups of HK-2 cells. Nuclei were counterstained with DAPI (blue). Scale bar: 200 μm. Quantification of the protein levels of **B** TGF-β1, **D** p-smad2/3 in HK-2 cells. **E** Western blot analysis of smad2/3, p-p38 MAPK, p38 MAPK and TGF-β1 expression in HK-2 cells. Quantitative analysis the protein levels of **F** TGF-β1, **G**, **H** smad2/3, **I** p-p38 MAPK and **J** p38 MAPK in HK-2 cells. Reverse transcription-quantitative PCR analysis of *TGF-β1* mRNA expression **K** in HK-2 cells. n  = 3. *P  < 0.05 vs. HK-2; ^#^P  < 0.05 vs. Model; ^&^P  < 0.05 vs. SIS3

As presented by immunofluorescence staining, the protein levels of TGF-β1 and p-smad2/3 were significantly increased in the model group compared with the HK-2 group. Conversely, the protein levels of TGF-β1 in the EGb and SIS3 groups and p-smad2/3 in the EGb, SIS3 and EGb  +  SIS3 groups were decreased compared with those in the model group (Fig. 5A, C).

Western blot analysis confirmed that the protein levels of TGF-β1, smad2/3, p38 MAPK and p-p38 MAPK were significantly increased in the model group compared with the HK-2 group. Additionally, compared with the model group, the protein levels of TGF-β1, smad2/3, and p-p38 MAPK were significantly decreased in the EGb, SIS3 and EGb  +  SIS3 groups, and the protein level of p38 MAPK was significantly decreased in the EGb group (Fig. 5).

RT–qPCR analysis demonstrated that the mRNA level of *TGF-β1* was significantly increased in the model group compared with the HK-2 group, while it was significantly decreased in the EGb, SIS3 and EGb  +  SIS3 groups compared with the model group (Fig. 5K).

## Discussion

Previous studies have demonstrated that rat renal lesions induced by cisplatin may be used as an experimental model for the induction of renal tubular injury and interstitial fibrosis [26, 27]. Cisplatin treatment increased the levels of α-SMA and induced aberrant collagen deposition in renal tissues, leading to tubular injury and interstitial fibrosis [28, 29]. In the present study, the levels of Scr, BUN and urinary NAG, as well as the tubular injury scores, were significantly increased following exposure to a single dose of cisplatin compared with those in the control group. These results suggested that cisplatin induced kidney damage. Additionally, Masson’s trichrome staining indicated that the renal interstitial tissues exhibited fibroplasia, and immunohistochemical analysis demonstrated that the renal α-SMA and Col I levels were significantly increased following exposure to cisplatin compared with those in the control group. These results demonstrated that a single dose of cisplatin (5 mg/kg body weight), which was similar to the dose commonly used in clinical applications [30], induced renal interstitial fibrosis in a rat model. These results also suggested that the incidence of CRIF may be high. However, the mechanism of CRIF is unclear, and effective treatments for CRIF are lacking.

EMT of renal tubular epithelial cells is considered a major mechanism of CRIF [31]. TGF-β1, an important soluble factor in the induction of fibrosis [32], can be significantly increased in rat CRIF [33]. Previous reports have shown that the levels of TGF-β1 and Smad are increased in cisplatin-treated rats [34] and that the p-p38 MAPK level is increased in HK-2 cells after cisplatin-induced EMT [34]. Our previous study found that inhibition of TGF-β1 and p38 MAPK levels in renal tissues could reduce renal interstitial fibrosis after cisplatin-induced acute renal injury in rats [5]. However, the roles and relationships of these three cytokines in the progression of CRIF and renal tubular epithelial cell EMT are unclear. In our study, the protein levels of TGF-β1, smad2/3, p-smad2/3 and p-p38 MAPK, as well as the ratio of p-p38 MAPK/p38 MAPK and the mRNA level of *p38 MAPK,* were significantly increased in renal tissues after rat exposure to cisplatin. Additionally, the changes in these indicators were consistent with the decreased renal function and increased levels of α-SMA and COL I in rat kidneys. These results suggested that TGF-β1, smad2/3 and p38 MAPK may mediate CRIF in rats.

In an in vitro study, the protein levels of α-SMA, vimentin, COL-I, CTGF and TIMP-1 were significantly increased, while E-cadherin protein levels and the ratio of MMP-1/TIMP-1 were significantly decreased in HK-2 cells after exposure to cisplatin. These findings suggested that cisplatin induced EMT in HK-2 cells. Moreover, the protein levels of TGF-β1, smad2/3, p-smad2/3, p38 MAPK, and p-p38 MAPK and the mRNA level of *TGF-β1* in HK-2 cells were also markedly elevated after exposure to cisplatin. Importantly, SIS3 (a specific inhibitor of smad3) significantly reversed the cisplatin-induced EMT of HK-2 cells and the changes in related indicators, especially the protein levels of smad2/3, p-smad2/3, p-p38 MAPK and TGF-β1 and the mRNA level of *TGF-β1*. These data confirmed that smad3 could regulate the expression of TGF-β1 and p38 MAPK and the cisplatin-induced EMT of HK-2 cells. Combined with the above animal experimental results, cisplatin induced EMT of renal tubular epithelial cells by upregulating Smad3/TGF-β1 and Smad3/p38 MAPK, thereby inducing renal interstitial fibrosis.

EGb is a traditional Chinese herbal medicine that includes a variety of active ingredients, such as flavonoids and terpene lactones, and has been widely used in clinical practice for a number of years [35]. EGb has attracted attention due to its treatment applications for a range of diseases, such as Alzheimer’s disease, cerebral insufficiency, coronary heart disease and renal ischemia–reperfusion injury [14, 36]. According to previous studies, EGb not only prevented renal fibrosis in rats with diabetic nephropathy [37] but also improved acute kidney injury induced by cisplatin in rat and cell models [29]. Our previous study also demonstrated that EGb could protect against acute renal injury induced by cisplatin in a rabbit model [19] and alleviate CRIF in rats by inhibiting renal apoptosis [5]. In the present study, EGb treatment significantly decreased the cisplatin-induced high levels of Scr, BUN and urinary NAG in rats. Masson's trichrome staining demonstrated that EGb significantly decreased the degree of interstitial fibrosis in the kidney induced by cisplatin. EGb treatment also significantly decreased the cisplatin-induced high levels of α-SMA and Col I in renal tissues. Additionally, in an in vitro experiment, EGb significantly decreased the protein levels of α-SMA, vimentin, COL-I, CTGF, and TIMP-1 while significantly increasing the E-cadherin protein level and the ratio of MMP-1/TIMP-1 induced by cisplatin in HK-2 cells. These results suggested that EGb could attenuate CRIF by inhibiting the EMT of renal tubular epithelial cells. However, the exact molecular mechanisms remain to be elucidated.

In our study, TGF-β1 expression in the kidney was significantly upregulated after cisplatin treatment compared with the control group. There is also a report in the literature that in a mouse renal fibrosis model induced by unilateral ureteral obstruction, the level of TGF-β1 protein in kidney tissue is increased, similar to our result [38]. In addition, TGF-β1 protein expression was significantly decreased in EGb-treated rats compared with cisplatin-treated rats. In the same way. One study documented the suppression of gene expression in kidney TGF-β after the use of EGb in a rat kidney injury model induced by methotrexate [39]. Some reports suggest that EGb also inhibits the activation of hepatic stellate cells and improves liver fibrosis and liver function partially by inhibiting the p38 MAPK pathway [17]. EGb delayed the development of glomerular sclerosis in rats with diabetic nephropathy by reducing smad2/3 expression [40]. In the present study, EGb significantly decreased the protein levels of TGF-β1, smad2/3, p-smad2/3, p38 MAPK and p-p38 MAPK, as well as the p-p38 MAPK/p38 MAPK ratio and the *p38 MAPK* mRNA level in rat kidneys induced by cisplatin. Additionally, EGb decreased the protein levels of the above indicators, as well as the mRNA level of *TGF-β1* in HK-2 cells induced by cisplatin. These data suggested that EGb inhibited the EMT of renal tubular epithelial cells by downregulating p38 MAPK, TGF-β1 and Smads and may be an important mechanism of EGb protection against CRIF.

In conclusion, combined with the results of the above animal and cell experiments, EGb inhibited cisplatin-induced EMT of renal tubular epithelial cells by downregulating the smad3/TGF-β1 and smad3/p38 MAPK pathways and ultimately effectively ameliorated CRIF.

This paper presented innovations. First, the roles and relationships of smad3, TGF-β1 and p38 MAPK in the cisplatin-induced EMT of renal tubular epithelial cells were elucidated in this paper. Second, this paper also demonstrated that EGb inhibited cisplatin-induced EMT of renal tubular epithelial cells by downregulating the smad3/TGF-β1 and smad3/p38 MAPK pathways and ultimately effectively ameliorated CRIF.

There were certain limitations to the present study. Only a limited number of indicators of kidney function were detected, namely, BUN, Scr and urinary NAG, which have been proposed to be sensitive and robust indicators of kidney damage in rodents and humans [41]. However, the assessment of kidney function may be more accurate if other indicators, such as urinary creatinine, albumin and 24-h volume, are tested at the same time.

## Conclusions

In short, our findings suggested that EGb inhibited cisplatin-induced EMT of renal tubular epithelial cells by downregulating the smad3/TGF-β1 and smad3/p38 MAPK pathways and ultimately effectively ameliorated CRIF. Taken together, we suggested that EGb has beneficial effects on alleviating renal interstitial fibrosis and could be a potential drug for renal failure.

## Supplementary Information

**Additional file 1: Effects of EGb on p-smad2/smad2, p-smad3/smad3, the phosphorylated/total protein p-p38 MAPK/p38 MAPK. Figure S1.** The effect of EGb on the protein expression levels of smad2, p-smad2, smad3, p-smad3, p-smad2/smad2, p-smad3/smad3 in rat kidney tissue. Western blot analysis of **A** smad2/3, **B** p-smad2/3 expression in rat kidney tissues. Quantitative analysis the protein levels of **C** smad2, **D** smad3, **E** p-smad2, **F** p-smad3, **G** p-smad2/smad2, **H** p-smad3/smad3 in renal tissues. n  = 9. *P < 0.05 vs. control; ^#^P < 0.05 vs. cisplatin; ^&^P < 0.05 vs. cisplatin + M-EGb. *MAPK* mitogen-activated protein kinase; *p-* phosphorylated; *L* low-dose; *M* medium-dose; *H* high-dose; *EGb* *Ginkgo* *biloba* leaf extract. **Figure S2. **The effect of EGb on the protein levels of smad2, p-smad2, smad3, p-smad3, p-smad2/smad2, p-smad3/smad3 in HK-2 cells. Western blot analysis of **A** smad2/3, **B** p-smad2/3 expression in HK-2 cells. Quantitative analysis the protein levels of **C** smad2, **D** smad3, **E** p-smad2, **F** p-smad3, **G** psmad2/smad2, **H** p-smad3/smad3 in HK-2 cells. n  = 3. *P < 0.05 vs. HK-2; ^#^P < 0.05 vs. Model; ^&^P < 0.05 vs. SIS3. **Figure S3. **The effect of EGb on the protein levels of p-p38 MAPK, p38 MAPK and p-p38 MAPK/p38 MAPK in HK-2 cells. Western blot analysis of **A** p-p38 MAPK, **B** p38 MAPK expression in HK-2 cells. Quantitative analysis the protein levels of **C** p-p38 MAPK, **D** p38 MAPK, **E** p-p38 MAPK/p38 MAPK in HK-2 cells. n  = 3. *P < 0.05 vs. HK-2; ^#^P < 0.05 vs. Model; ^&^P < 0.05 vs. SIS3.

**Additional file 2: Cell proliferation experiment. Figure S1.** The effect of EGb on HK2 cells proliferation and activity. n  = 3. *P < 0.05 vs. control.

## Data Availability

The data and materials in this study are available from the corresponding author on reasonable request.

## Abbreviations

EGb: *Ginkgo* *biloba* leaf extract
Scr: Serum creatinine
BUN: Blood urea nitrogen
NAG: N-acetyl-β-D- glucosaminidase
α-SMA: α-Smooth muscle actin
Col I: Type I collagen
CRIF: Renal interstitial fibrosis
EMT: Epithelial-mesenchymal transition
TGF-β1: Transforming growth factor β1
MAPK: Mitogen-activated protein kinase
MEM: Minimum essential medium
β-actin: Beta-actin
HK-2: Human renal tubular epithelial
FBS: Fetal bovine serum
H&E: Hematoxylin and eosin
RT-q: Reverse transcription-quantitative
DAB: Diaminobenzidine
IOD: Integrated optical density

## References

[1] Shen DW, Pouliot LM, Hall MD, Gottesman MM (2012). Cisplatin resistance: a cellular self-defense mechanism resulting from multiple epigenetic and genetic changes. Pharmacol Rev.

[2] Pabla N, Dong Z (2008). Cisplatin nephrotoxicity: mechanisms and renoprotective strategies. Kidney Int.

[3] Yu CC, Chien CT, Chang TC (2016). M2 macrophage polarization modulates epithelial-mesenchymal transition in cisplatin-induced tubulointerstitial fibrosis. Biomedicine.

[4] Qi W, Chen X, Poronnik P, Pollock CA (2006). The renal cortical fibroblast in renal tubulointerstitial fibrosis. Int J Biochem Cell Biol.

[5] Liang T, Wei C, Lu S, Qin M, Qin G, Zhang Y (2021). Ginaton injection alleviates cisplatin-induced renal interstitial fibrosis in rats via inhibition of apoptosis through regulation of the p38MAPK/TGF-beta1 and p38MAPK/HIF-1alpha pathways. Biomed Rep.

[6] Lu Q, Chen YB, Yang H, Wang WW, Li CC, Wang L (2019). Inactivation of TSC1 promotes epithelial-mesenchymal transition of renal tubular epithelial cells in mouse diabetic nephropathy. Acta Pharmacol Sin.

[7] Desmouliere A, Darby IA, Gabbiani G (2003). Normal and pathologic soft tissue remodeling: role of the myofibroblast, with special emphasis on liver and kidney fibrosis. Lab Invest.

[8] Zeisberg M, Bonner G, Maeshima Y, Colorado P, Müller GA, Strutz F (2001). Renal fibrosis: collagen composition and assembly regulates epithelial-mesenchymal transdifferentiation. Am J Pathol.

[9] Yamamoto E, Izawa T, Juniantito V, Kuwamura M, Sugiura K, Takeuchi T (2010). Involvement of endogenous prostaglandin E2 in tubular epithelial regeneration through inhibition of apoptosis and epithelial-mesenchymal transition in cisplatin-induced rat renal lesions. Histol Histopathol.

[10] Liu C, Chen F, Han X, Xu H, Wang Y (2014). Role of TGF-β1p38 MAPK pathway in hepatitis B virus-induced tubular epithelial-myofibroblast transdifferentiation. Int J Clin Exp Pathol.

[11] Pan MM, Zhang MH, Ni HF, Chen JF, Xu M, Phillips AO (2013). Inhibition of TGF-beta1/Smad signal pathway is involved in the effect of *Cordyceps**sinensis* against renal fibrosis in 5/6 nephrectomy rats. Food Chem Toxicol.

[12] Sun YZ, Gu XH, Yang XQ, Zhu WQ, Guo LP, Huang LQ, Pan YZ (2007). Study on dynamical changes of *Ginkgo**biloba* resources in Pizhou city of Jiangsu Province base on RS and GIS. Zhongguo Zhong Yao Za Zhi.

[13] Yao X, Zhou G, Tang Y, Guo S, Qian D, Duan JA (2015). HILIC-UPLC-MS/MS combined with hierarchical clustering analysis to rapidly analyze and evaluate nucleobases and nucleosides in *Ginkgo* biloba leaves. Drug Test Anal.

[14] Fu LM, Li JT (2011). A systematic review of single Chinese herbs for Alzheimer’s disease treatment. Evid Based Complement Alternat Med.

[15] Kandiah N, Ong PA, Yuda T, Ng LL, Mamun K, Merchant RA (2019). Treatment of dementia and mild cognitive impairment with or without cerebrovascular disease: expert consensus on the use of *Ginkgo**biloba* extract, EGb 761((R)). CNS Neurosci Ther.

[16] Liu S-Q, Yu J-P, Chen H-L, Luo H-S, Chen S-M, Yu H-G (2006). Therapeutic effects and molecular mechanisms of *Ginkgo**biloba* extract on liver fibrosis in rats. Am J Chin Med.

[17] Wang Y, Wang R, Wang Y, Peng R, Wu Y, Yuan Y (2015). *Ginkgo**biloba* extract mitigates liver fibrosis and apoptosis by regulating p38 MAPK, NF-kappaB/IkappaBalpha, and Bcl-2/Bax signaling. Drug Des Dev Ther.

[18] Li Y, Xiong Y, Zhang H, Li J, Wang D, Chen W (2017). *Ginkgo**biloba* extract EGb761 attenuates brain death-induced renal injury by inhibiting pro-inflammatory cytokines and the SAPK and JAK-STAT signalings. Sci Rep.

[19] Yang YF, Lao S, Luo M, Zeng J (2011). Dynamic observation of the protective effect of *Ginkgo**biloba* extract on cisplatin kidney damage in rabbits. Lishizhen Med Mater Med.

[20] Deng YK, Wei F, Zhang DG (2006). Brain protective effects of *Ginkgo**biloba* leaf extract (ginaton) in patients undergoing hypothermic cardiopulmonary bypass. Chin J Integr Tradit West Med.

[21] Moustafa FE, Sobh MA, Abouelkheir M, Khater Y, Mahmoud K, Saad MA (2016). Study of the effect of route of administration of mesenchymal stem cells on cisplatin-induced acute kidney injury in Sprague Dawley rats. Int J Stem Cells.

[22] Sisi LU, Zhong X, Yang Y, Zou X, Liang X, Cai G (2018). Effects of single dose of cisplatin on renal interstitial fibrosis indicators in rats. China Pharm.

[23] Yamate J, Tatsumi M, Nakatsuji S, Kuwamura M, Kotani T, Sakuma S (1995). Immunohistochemical observations on the kinetics of macrophages and myofibroblasts in rat renal interstitial fibrosis induced by cis-diamminedichloroplatinum. J Comp Pathol.

[24] Kawai Y, Satoh T, Hibi D, Ohno Y, Kohda Y, Miura K (2009). The effect of antioxidant on development of fibrosis by cisplatin in rats. J Pharmacol Sci.

[25] Livak KJ, Schmittgen TD (2001). Analysis of relative gene expression data using real-time quantitative PCR and the 2[-Delta Delta C(T)] Method. Methods.

[26] Yano R, Golbar HM, Izawa T, Sawamoto O, Kuwamura M, Yamate J (2015). Participation of bone morphogenetic protein (BMP)-6 and osteopontin in cisplatin (CDDP)-induced rat renal fibrosis. Exp Toxicol Pathol.

[27] Liu Q, Hu S, He Y, Zhang J, Zeng X, Gong F (2017). The protective effects of Zhen-Wu-Tang against cisplatin-induced acute kidney injury in rats. PLoS ONE.

[28] Yuasa T, Yano R, Izawa T, Kuwamura M, Yamate J (2014). Calponin expression in renal tubulointerstitial fibrosis induced in rats by cisplatin. J Toxicol Pathol.

[29] Song J, Liu D, Feng L, Zhang Z, Jia X, Xiao W (2013). Protective effect of standardized extract of *Ginkgo**biloba* against cisplatin-induced nephrotoxicity. Evid Based Complement Alternat Med.

[30] Sato A, Kurihara M, Matsukawa M, Shimada K, Yamazaki T, Nakamachi M (2001). Preliminary study of fortnightly irinotecan hydrochloride plus cisplatin therapy in patients with advanced gastric and colorectal cancer. Cancer Chemother Pharmacol.

[31] Song Y, Lv S, Wang F, Liu X, Cheng J, Liu S (2020). Overexpression of BMP7 reverses TGFbeta1induced epithelialmesenchymal transition by attenuating the Wnt3/betacatenin and TGF-beta1/Smad2/3 signaling pathways in HK2 cells. Mol Med Rep.

[32] Li J, Cen B, Chen S, He Y (2016). MicroRNA-29b inhibits TGF-beta1-induced fibrosis via regulation of the TGF-beta1/Smad pathway in primary human endometrial stromal cells. Mol Med Rep.

[33] Park J-S, Jo CH, Kim S, Kim G-H (2013). Acute and chronic effects of dietary sodium restriction on renal tubulointerstitial fibrosis in cisplatin-treated rats. Nephrol Dial Transpl.

[34] Park JW, Cho JW, Joo SY, Kim CS, Choi JS, Bae EH (2012). Paricalcitol prevents cisplatin-induced renal injury by suppressing apoptosis and proliferation. Eur J Pharmacol.

[35] Zheng B, Teng L, Xing G, Bi Y, Yang S, Hao F (2015). Proliposomes containing a bile salt for oral delivery of *Ginkgo**biloba* extract: formulation optimization, characterization, oral bioavailability and tissue distribution in rats. Eur J Pharm Sci.

[36] Sun M, Chai L, Lu F, Zhao Y, Li Q, Cui B (2018). Efficacy and safety of *Ginkgo**biloba* pills for coronary heart disease with impaired glucose regulation: study protocol for a series of N-of-1 randomized, double-blind, placebo-controlled trials. Evid Based Complement Alternat Med.

[37] Lu Q, Zuo WZ, Ji XJ, Zhou YX, Liu YQ, Yao XQ (2015). Ethanolic *Ginkgo**biloba* leaf extract prevents renal fibrosis through Akt/mTOR signaling in diabetic nephropathy. Phytomedicine.

[38] Geng XQ, Ma A, He JZ, Wang L, Jia YL, Shao GY (2020). Ganoderic acid hinders renal fibrosis via suppressing the TGF-beta/Smad and MAPK signaling pathways. Acta Pharmacol Sin.

[39] Sherif IO, Al-Shaalan NH, Sabry D (2019). *Ginkgo**biloba* extract alleviates methotrexate-induced renal injury: new impact on PI3K/Akt/mTOR signaling and MALAT1 expression. Biomolecules.

[40] Wang JY, Yin XX, Wu YM, Tang DQ, Gao YY, Wan MR (2006). *Ginkgo**biloba* extract suppresses hypertrophy and extracellular matrix accumulation in rat mesangial cells. Acta Pharmacol Sin.

[41] Vaidya VS, Waikar SS, Ferguson MA, Collings FB, Sunderland K, Gioules C (2008). Urinary biomarkers for sensitive and specific detection of acute kidney injury in humans. Clin Transl Sci.

