# Supplementary material for: Ginkgobiloba leaf extract mitigates cisplatin-induced chronic renal interstitial fibrosis by inhibiting the epithelial-mesenchymal transition of renal tubular epithelial cells mediated by the Smad3/TGF-β1 and Smad3/p38 MAPK pathways
Source: Chin Med. 2022 Feb 21;17:25. doi: 10.1186/s13020-022-00574-y (PMC8862328; doi:10.1186/s13020-022-00574-y)
Supplement: Supplementary file 1 — Additional file 1: Effects of EGb on p-smad2/smad2, p-smad3/smad3, the phosphorylated/total protein p-p38 MAPK/p38 MAPK. Figure S1. The effect of EGb on the protein expression levels of smad2, p-smad2, smad3, p-smad3, p-smad2/smad2, p-smad3/smad3 in rat kidney tissue. Western blot analysis of A smad2/3, B p-smad2/3 expression in rat kidney tissues. Quantitative analysis the protein levels of C smad2, D smad3, E p-smad2, F p-smad3, G p-smad2/smad2, H p-smad3/smad3 in renal tissues. n = 9. *P < 0.05 vs. control; #P < 0.05 vs. cisplatin; &P < 0.05 vs. cisplatin + M-EGb. MAPK mitogen-activated protein kinase; p- phosphorylated; L low-dose; M medium-dose; H high-dose; EGb Ginkgo biloba leaf extract. Figure S2. The effect of EGb on the protein levels of smad2, p-smad2, smad3, p-smad3, p-smad2/smad2, p-smad3/smad3 in HK-2 cells. Western blot analysis of A smad2/3, B p-smad2/3 expression in HK-2 cells. Quantitative analysis the protein levels of C smad2, D smad3, E p-smad2, F p-smad3, G psmad2/smad2, H p-smad3/smad3 in HK-2 cells. n = 3. *P < 0.05 vs. HK-2; #P < 0.05 vs. Model; &P < 0.05 vs. SIS3. Figure S3. The effect of EGb on the protein levels of p-p38 MAPK, p38 MAPK and p-p38 MAPK/p38 MAPK in HK-2 cells. Western blot analysis of A p-p38 MAPK, B p38 MAPK expression in HK-2 cells. Quantitative analysis the protein levels of C p-p38 MAPK, D p38 MAPK, E p-p38 MAPK/p38 MAPK in HK-2 cells. n = 3. *P < 0.05 vs. HK-2; #P < 0.05 vs. Model; &P < 0.05 vs. SIS3. [file 13020_2022_574_MOESM1_ESM.docx]

**Effects of EGb on pSmad2/Smad2, pSmad3/Smad3, the phosphorylated/total protein p-p38MAPK/p38MAPK**

***1.Effects of EGb on the levels of smad2, p-smad2, smad3, p-smad3, p-smad2/smad2andp-smad3/smad3in renal tissues from rats treated with cisplatin.***

In vivo experiments, as shown in Figures 1C (Figure 3, E in the manuscript) and 1E (Figure 3, G in the manuscript), compared with the Control group, the p-smad2 and smad2 protein levels in the Cisplatin group were significantly increased. Additionally, as shown in Figure 1G, compared with the Control group, the ratio of p-smad2/smad2 in the Cisplatin group was significantly increased. These results indicate that Cisplatin increased the p-smad2 protein level more than the smad2 protein level.

As shown in Figures 1C and 1E, compared with the Cisplatin group, the smad2 and p-smad2 protein levels in the Cisplatin+L-EGb group were not different. Additionally, as shown in Figure 1G, compared with the Cisplatin group, there was no difference in the ratio of p-smad2/smad2 in the Cisplatin+L-EGb group. These results indicate that L-EGb did not change the protein levels of p-smad2 and smad2.

As shown in Figure 1C and 1E, compared with the Cisplatin group, there was no difference in the smad2 protein level of the Cisplatin+M-EGb group, but the p-smad2 protein level was significantly reduced. Additionally, as shown in Figure 1G, there was no difference in the ratio of p-smad2/smad2 between Cisplatin group and Cisplatin+M-EGb.

It can be seen from Figures 1C and 1E that H-EGb significantly reduced Cisplatin-induced elevated smad2 and p-smad2 protein levels. Additionally, as shown in Figure 1G, the ratio of p-smad2/smad2 in the Cisplatin+H-EGb group was significantly higher than that in the Cisplatin group. Considering that cisplatin increases the level of p-smad2 protein more than the increase of smad2 protein level, these results indicate that the reduction of p-smad2 protein level by H-EGb is less than the reduction of smad2 protein level.

In the in vivo experiment, as shown in Figure 1D (Figure 3, F of the manuscript) and 1F (Figure 3, H of the manuscript), compared with the Control group, the smad3 and p-smad3 protein levels of the Cisplatin group were significant increased. Additionally, as shown in Figure 1H, compared with the Control group, the ratio of p-smad3/smad3 in the Cisplatin group was significantly increased. These results indicate that the increase in p-smad3 protein level was greater than the increase in smad3 protein level.

As shown in Figures 1D and 1F, compared with the Cisplatin group, the smad3 protein level of the Cisplatin+L-EGb group was significantly reduced, and there was no difference in the p-smad3 protein level. Additionally, as shown in Figure 1H, compared with the Cisplatin group, Cisplatin+L-EGb significantly increased the ratio of p-smad3/smad3, which was consistent with the results of Figures 1D and 1F, indicating that L-EGb reduces smad3.

As shown in Figures 1D and 1F, the smad3 protein level was no difference between Cisplatin group and Cisplatin+M-EGb group, while the p-smad3 protein level was significantly reduced in Cisplatin+M-EGb group. Additionally, as shown in Figure 1H, the ratio of p-smad3/smad3 was no difference between Cisplatin group and Cisplatin+M-EGb group.

As shown in Figures 1D and 1F, compared with the Cisplatin group, the smad3 and p-smad3 protein levels of the Cisplatin+H-EGb group were significantly reduced. Additionally, as shown in Figure 1H, the ratio of p-smad3/smad3 was no difference between Control group and Cisplatin+H-EGb group. These results indicate that H-EGb reduced p-smad3 protein level as much as it reduced smad3 protein level.
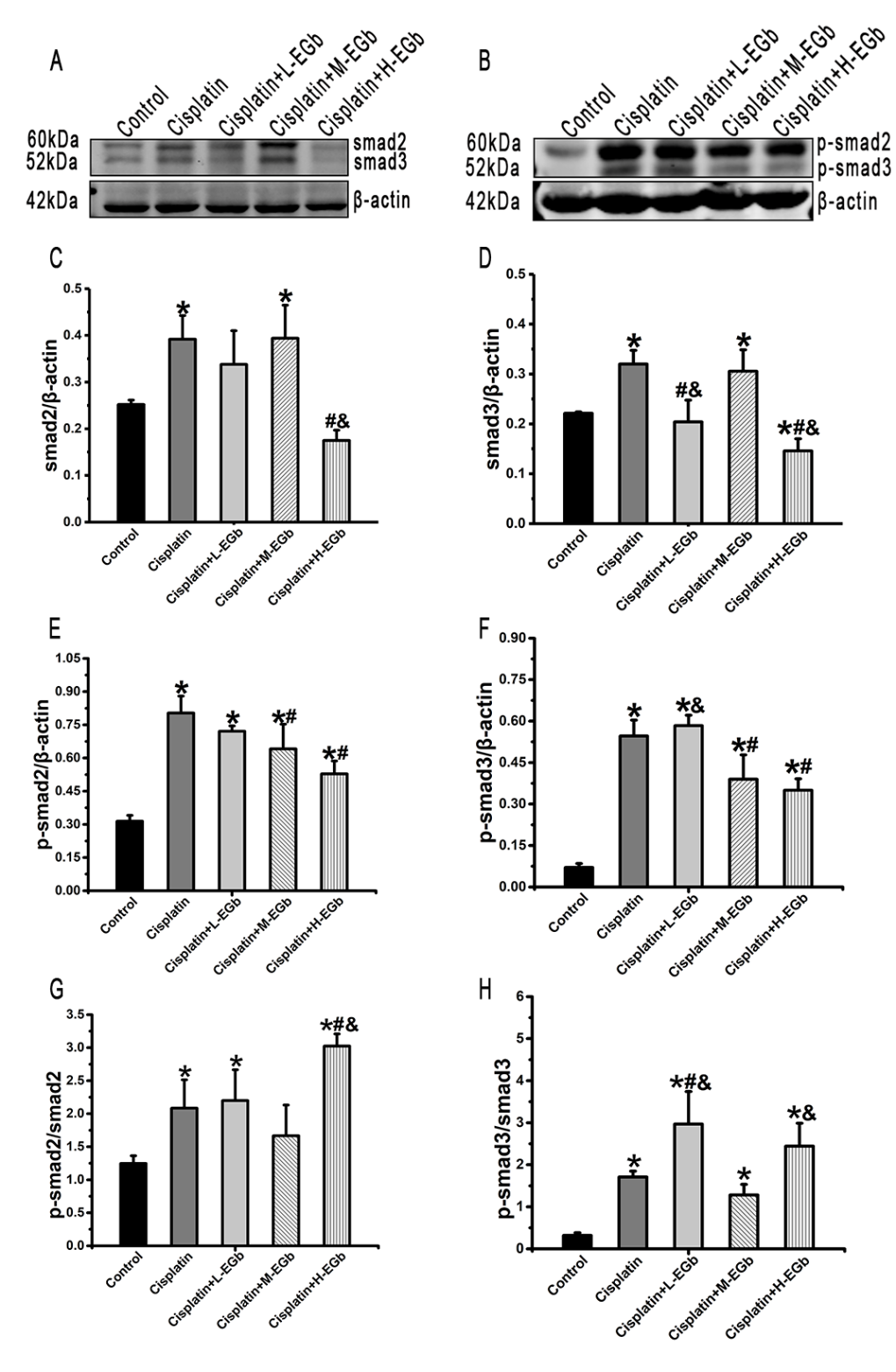


**Figure 1.** The effect of EGb on the protein expression levels of smad2, p-smad2, smad3, p-smad3, p-smad2/smad2, p-smad3/smad3 in rat kidneytissue. Western blot analysis of (A) smad2/3, (B) p-smad2/3 expression in rat kidney tissues.Quantitative analysis the protein levels of(C) smad2, (D) smad3, (E) p-smad2, (F) p-smad3, (G) p-smad2/smad2,(H) p-smad3/smad3 in renal tissues. n=9. *P<0.05 vs. control; ^#^P<0.05 vs. cisplatin; ^&^P < 0.05 vs. cisplatin + M-EGb. MAPK: mitogen-activated protein kinase; p-: phosphorylated; L: low-dose; M: medium-dose; H: high-dose; EGb: Ginkgo biloba leaf extract.

***2. Effects of EGb on the levels of smad2, p-smad2, smad3, p-smad3, p-smad2/smad2andp-smad3/smad3 in HK-2 cells injured by cisplatin*.**

In the in vitro experiment, as shown in Figure 2C (Figure 5, G of the manuscript) and 2E, compared with the HK-2 group, the smad2 protein level in the Model group was significantly increased, while the p-smad2 protein level was no change. Additionally, as shown in Figure 2G, compared with the HK-2 group, the ratio of p-smad2/smad2 in the Model group was significantly reduced, which was consistent with the results of Figures 2C and 2E, indicating that cisplatin significantly increased smad2 protein level.

As shown in Figures 2C and 2E, compared with the Model group, the smad2 protein level in the EGb group was significantly reduced, while the p-smad2 protein level was no change. Additionally, as shown in Figure 2G, compared with the Model group, the ratio of p-smad2/smad2 in the EGb group significantly increased, which was consistent with the results of Figures 2C and 2E, indicating that EGb significantly reduced smad2 protein level.

As shown in Figure 2C and 2E, compared with the Model group, the smad2 protein level of the SIS3 group was significantly reduced, while the p-smad2 protein level was no change. Additionally, as shown in Figure 2G, the ratio of p-smad2/smad2 was no difference between the Model group and the SIS3 group.

As shown in Figure 2C and 2E, compared with the Model group, the p-smad2 protein level was significantly increased in the EGb+SIS3 group, while the smad2 protein level was no change. Additionally, as shown in Figure 2G, compared with the Model group, the p-smad2/smad2 ratio in the EGb+SIS3 group was significantly increased, which was consistent with the results of Figures 2C and 2E, indicating that EGb + SIS3 significantly increased smad2 protein level.

In the in vitro experiments, as shown in Figure 2D (Figure 5, H of the manuscript) and 2F, compared with the HK-2 group, the smad3 protein level in the Model group was significantly increased, while the p-smad3 protein level was no change. Additionally, as shown in Figure 2H, compared with HK-2, the ratio of p-smad3/smad3 in the Model group was significantly reduced, which was consistent with the results of Figures 2D and 2F, indicating that cisplatin significantly increased smad3 protein level.

As shown in Figure 2D and 2F, compared with the Model group, the smad3 protein level of the EGb group was significantly reduced, while the p-smad3 protein level was no change. Additionally, as shown in Figure 2H, compared with the Model group, the ratio of p-smad3/smad3 in the EGb group was significantly increased, which was consistent with the results of Figures 2D and 2F, indicating that EGb significantly reduced smad3 protein level.

As shown in Figure 2D and 2F, compared with the Model group, the smad3 protein level in the SIS3 group was significantly reduced, while the p-smad3 protein level was no change. Additionally, as shown in Figure 2H, compared with the Model group, the ratio of p-smad3/smad3 in the SIS3 group was significantly increased, which was consistent with the results of Figures 2D and 2F, indicating that SIS3 significantly reduced smad3 protein level.

As shown in Figure 2D and 2F, compared with the Model group, the smad3 protein level of the EGb+SIS3 group was significantly reduced, while the p-smad3 protein level was no change. Additionally, as shown in Figure 2H, compared with the Model group, the ratio of p-smad3/smad3 in the EGb+SIS3 group was significantly increased, which was consistent with the results of Figures 2D and 2F, indicating that EGb+SIS3 significantly reduced smad3 protein level.


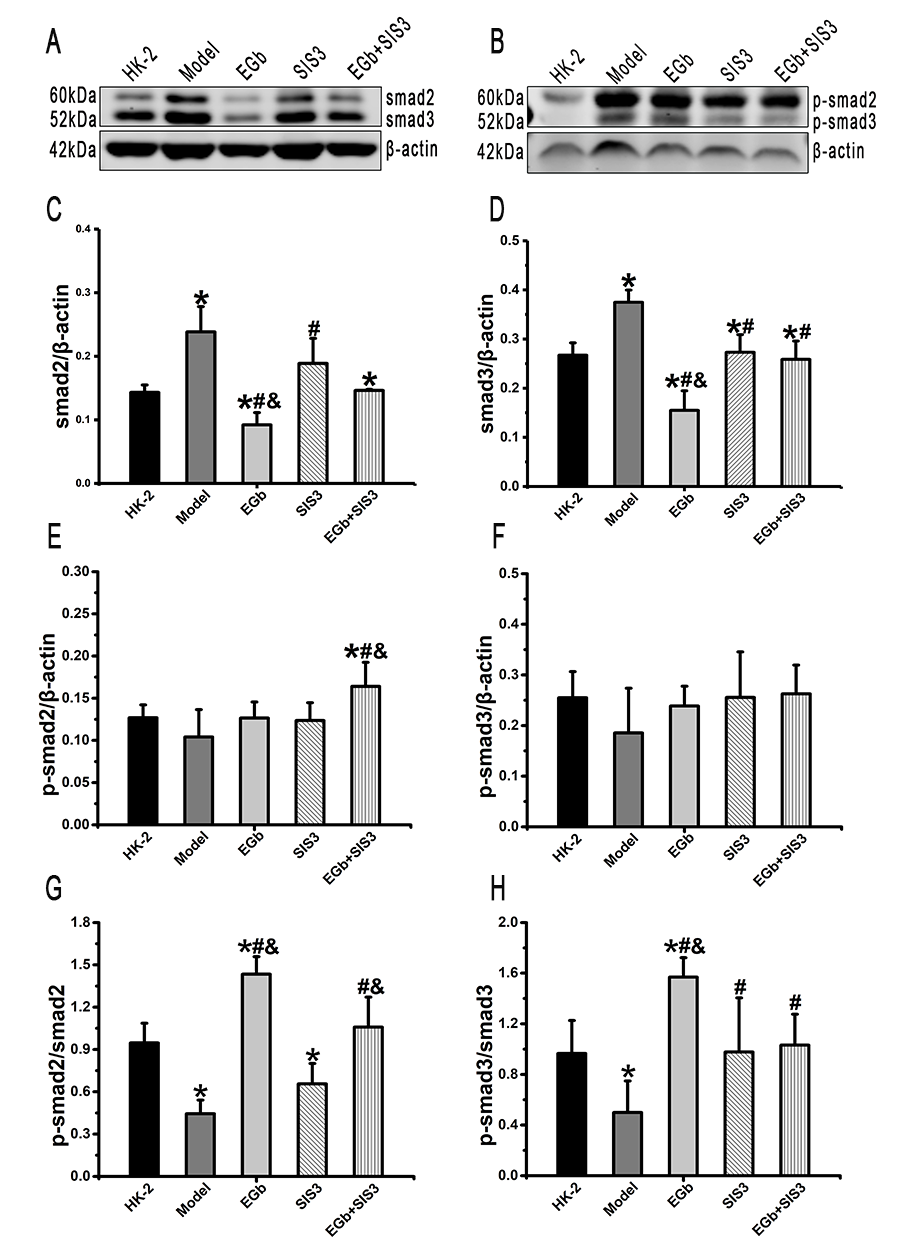


**Figure 2.** The effect of EGb on the protein levels of smad2, p-smad2, smad3, p-smad3, p-smad2/smad2, p-smad3/smad3in HK-2 cells. Western blot analysis of (A) smad2/3, (B) p-smad2/3expression in HK-2 cells. Quantitative analysis the protein levels of (C) smad2, (D) smad3, (E)p-smad2, (F) p-smad3,(G) psmad2/smad2, (H) p-smad3/smad3 in HK-2 cells. n=3. *P < 0.05 vs. HK-2; ^#^P < 0.05 vs. Model; ^&^P < 0.05 vs. SIS3.

***3. Effects of EGb on the levels of p-p38 MAPK,p38 MAPK andp-p38 MAPK/p38 MAPK in HK-2 cells injured by cisplatin*.**

In the in vitro experiments, as shown in Figure 3C (Figure 5, I of the manuscript) and 3D (Figure 5, J of the manuscript), compared with the HK-2 group, the protein levels of p-p38 MAPK and p38 MAPK in the CDDP group were significantly increased. Additionally, as shown in Figure 3E, compared with the HK-2 group, the ratio of p-p38 MAPK/p38 MAPK in the CDDP group was not different. These results indicate that cisplatin increased p38 MAPK protein level as much as it increased p-p38 MAPK protein level.

As shown in Figures 3C and 3D, compared with the Model group, the protein levels of p-p38 MAPK and p38MAPK in the EGb group were significantly reduced. Additionally, as shown in Figure 3E, the ratio of p-p38 MAPK/p38 MAPK was no difference between the Model group and the EGb group. These results indicate that EGb decreased p-p38MAPK protein level as much as it decreased p38 MAPK protein level.

As shown in Figure 3C and 3D, compared with the Model group, the p-p38 MAPK protein level in the SIS3 group and the EGb+SIS3 group was significantly reduced, while the p38MAPK protein level was no change. Additionally, as shown in Figure 3E, compared with the Model group, the ratio of p-p38 MAPK/p38 MAPK in the SIS3 group and the EGb+SIS3 group was significantly reduced , which was consistent with the results of Figures 3C and 3D, indicating that EGb reduces p-p38 MAPK.
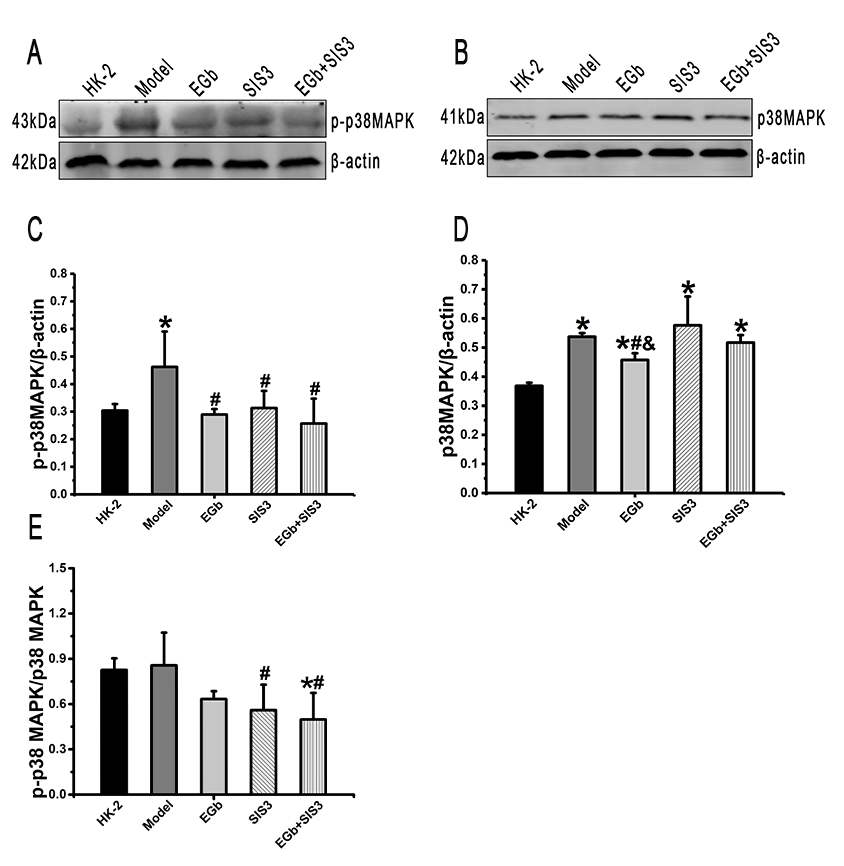


**Figure 3.** The effect of EGb on the protein levels of p-p38 MAPK,p38 MAPKand p-p38 MAPK/p38 MAPKin HK-2 cells. Western blot analysis of (A)p-p38 MAPK,(B)p38 MAPKexpression in HK-2 cells. Quantitative analysis the protein levels of (C) p-p38 MAPK, (D) p38 MAPK, (E)p-p38 MAPK/p38 MAPK in HK-2 cells. n=3. *P < 0.05 vs. HK-2; ^#^P < 0.05 vs. Model; ^&^P < 0.05 vs. SIS3.
