# Supplementary material for: Ginkgobiloba leaf extract mitigates cisplatin-induced chronic renal interstitial fibrosis by inhibiting the epithelial-mesenchymal transition of renal tubular epithelial cells mediated by the Smad3/TGF-β1 and Smad3/p38 MAPK pathways
Source: Chin Med. 2022 Feb 21;17:25. doi: 10.1186/s13020-022-00574-y (PMC8862328; doi:10.1186/s13020-022-00574-y)
Supplement: Supplementary file 2 — Additional file 2: Cell proliferation experiment. Figure S1. The effect of EGb on HK2 cells proliferation and activity. n = 3. *P < 0.05 vs. control. [file 13020_2022_574_MOESM2_ESM.docx]

**Cell proliferation experiment**

***1. Effect of EGb on HK2 cells proliferation and activity.***

The dosage of extract (350ug/ml) was derived from our pre-experimental results and literature reports. According to the references [1-6], we preliminarily determined a dose of EGb (175ug/ml), and then set a series of doses to conduct cytotoxicity tests (CCK8 assay) with this dose as the center and extending to both sides. As shown in Figure 1, the dose of extract (350ug/ml) was not toxic to renal tubular epithelial cells, and it was also consistent with the dose reported in the previous literature [1].





**Figure 1**. The effect of EGb on HK2 cells proliferation and activity. n=3. *P<0.05 vs. control.

References:

[1] Xu‚ X, Zheng‚ C, Tu‚ L, Zhao‚ G, Daowen Wang. Effect of Ginkgo Biloba Extract onTransdifferentiation of Transforming Growth Factor β1 Tubular Epithelial Cellsand Its Mechanism. Chinese Journal of Pathophysiology. 2008;24(11):2235-8.

[2] Di Meo F, Cuciniello R, Margarucci S, Bergamo P, Petillo O, Peluso G, et al. Ginkgo biloba Prevents Oxidative Stress-Induced Apoptosis Blocking p53 Activation in Neuroblastoma Cells. Antioxidants (Basel). 2020;9(4).

[3] Tingting Liu, Junzhong Zhang, Zhongqiu Chai, Gang Wang4, Naiqiang Cui, Bing Zhou1. EGb 761 Ginkgo biloba extract EGb 761–induced upregulation of LincRNA-p21 inhibits colorectal cancer metastasis by associating with EZH2. OncoTarget. 2017;8(53):91614-27.

[4] ZHENHuA Fu, SHIQuANLIu, MENGBIN QIN, JIEAN HuANG, CHuNYAN Xu, WENHoNG Wu, et al. NIKand IKKβbinding protein contributes to gastric cancer chemoresistance by promoting epithelialmesenchymal transition through the NF/κB signaling pathway. ONCOLOGY REPORTS 2018;39:2721-30.

[5] Chang L, Liu T, Chai Z, Jie S, Li Z, Liu M, et al. lincRNA-p21 Mediates the Anti-Cancer Effect of Ginkgo Biloba Extract EGb 761 by Stabilizing E-Cadherin Protein in Colon Cancer. Med SciMonit. 2018;24:9488-96.

[6] Choi SJ, Kim SW, Lee JB, Lim HJ, Kim YJ, Tian C, et al. Gingko biloba extracts protect auditory hair cells from cisplatin-induced ototoxicity by inhibiting perturbation of gap junctional intercellular communication. Neuroscience. 2013;244:49-61.
